# The megaproject paradox in urban street food areas: structural inertia and forced resilience in the Global South

**DOI:** 10.3389/fsoc.2026.1764890

**Published:** 2026-04-29

**Authors:** Sabar Aulia Rahman, Delmira Syafrini, Muhammad Rio Fariza, Bunga Dinda Permata, Ema Oktavia Ramadhani, Ardiani Fadila, Rania Alyasin, Fauziah Nadila Nelson Putri, Gusti Fauziah, Rayhan Adriansyah Permana

**Affiliations:** 1 Sociology Education Study Program, Faculty of Social Science, Universitas Negeri Padang, Padang, Indonesia; 2 Public Relations Study Program, School of Communication and Social Sciences, Telkom University, Bandung, Indonesia

**Keywords:** megaproject paradox, forced resilience, structural inertia, street food vendors, urban formalization, structuration theory, Global South, Bukittinggi

## Abstract

**Introduction:**

This study examines the paradox of state-led urban development through the “*Lambuang*” street food megaproject in Bukittinggi, Indonesia. Despite substantial investment, the project failed to achieve its economic goals and created a restrictive environment. Using Giddens’ Structuration Theory, this research analyzes how institutional structures and human agency interact amidst systemic failure.

**Methods:**

A qualitative case study was conducted, featuring in-depth interviews with 12 vendors and government representatives, field observations, and document analysis. Data were analyzed thematically to identify the interplay between structural constraints and agentic survival strategies.

**Results:**

The findings reveal “Structural Inertia,” where formal legality and infrastructure transformed from facilitators into coercive burdens. Formalization led to “Forced Resilience,” which is a condition where vendors remain in unproductive spaces not out of optimism, but due to legal entrapment and a lack of alternatives. This survival strategy paradoxically maintains the failing project’s existence while atomizing individual agency.

**Discussion:**

This research contributes to urban informality literature by challenging heroic resilience narratives in the Global South. It demonstrates that in failed development contexts, agency can reproduce dysfunctional structures through recursive survival practices. The study suggests that urban projects must prioritize functional adaptability and inclusive dialogue over rigid formalization to avoid sociological institutional traps. This intervention is crucial for understanding how legality and coercion shape economic adaptation in peripheral regions.

## 1 Introduction

Regional development is not merely rooted in technocratic dimensions but emerges from an ongoing dialectic between policy structures and the social actions of the communities embedded within them (Blažek and Květoň, 2023; Grillitsch and Sotarauta, 2020; Hawkins et al., 2016; Orczyk, 2025). Structuration theory emphasizes that structure not only constrains action but also simultaneously generates the resources that enable it through recurrent social practices. From this perspective, structure and agency are mutually constitutive (Giddens, 1984). Agency is not only shaped by rules but also reproduces those rules through everyday practices. Consequently, the success of development should not be measured solely through physical indicators but also by the extent to which communities are able to act as active subjects of development (Cook and Davíðsdóttir, 2021; Fukuda-Parr, 2003; Panagiotopoulos et al., 2024; Simonova, 2019). When policy structures become rigid, social agency is often pushed into adaptive survival situations, locally described as life, referring to the necessity of surviving under severe constraints. In such conditions, agency and structure interact in ways that can produce cycles of functional decline (Restrepo-Mieth et al., 2023). This tension between bureaucratic power and the capacity of social agency frequently becomes a fundamental cause of public project failure at the regional level (An and Bostic, 2021; Dasgupta and Kapur, 2020; Kim and Kang, 2024; Lazzarini et al., 2020).

This issue has global relevance, particularly in the context of Sustainable Development Goal 8, which promotes inclusive economic growth. Critiques by Sen (1999) and Stiglitz (2015) highlight that development policies often privilege structural actors while marginalizing small-scale economic participants. This dynamic is reflected in what has been described as the megaproject paradox (Gil, 2022), in which large-scale projects fail to generate sustainable economic spaces. Cases such as ghost markets in China and failed public markets in India illustrate how the absence of community participation in planning processes can cause physical spaces to lose their long-term social function (Liu et al., 2024; Sinha and Choudhury, 2023). In the Southeast Asian context, this phenomenon aligns with the concept of urban informality, whereby planning often fails because it creates conditions that overlook the organic economic logic of small-scale traders (Roy, 2005).

**Figure 1:**
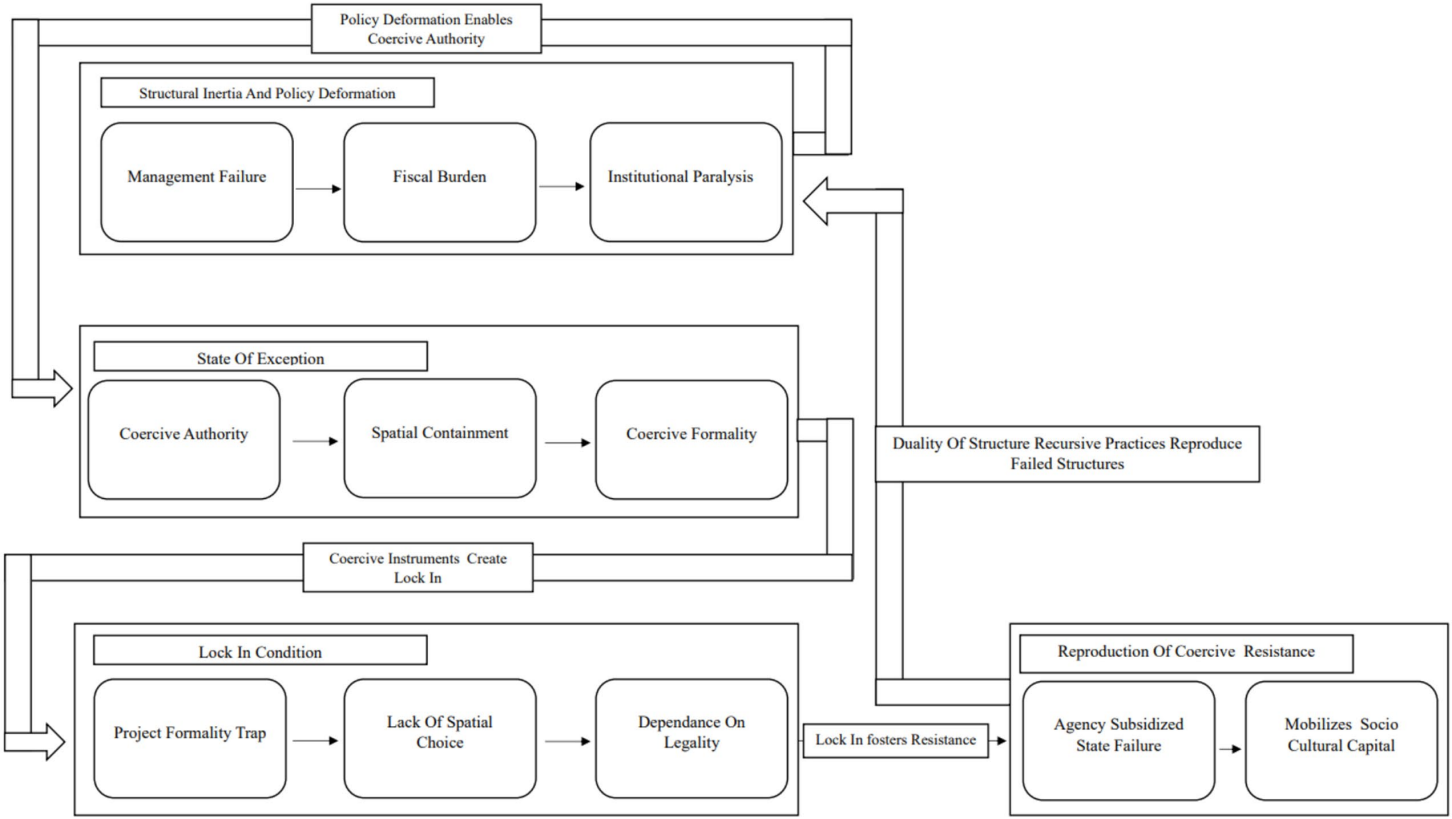
Conceptual model of the structuration of forced resilience in failed development projects. Source: First author findings, 2025.

This phenomenon is evident in the case of the *Lambuang* Street Food Area in Bukittinggi, West Sumatra, Indonesia. According to data from the Bukittinggi Office of Cooperatives, SMEs, and Trade 2025, the project required an investment of approximately 24.5 billion Indonesian Rupiah but ultimately encountered significant financial and social obstacles. The local government remains responsible for a long-term land lease valued at 9.7 billion Rupiah for the period 2022–2027, paid to the state-owned railway company that owns the land. Although daily management has been delegated to a traders’ association, the local government, through the Regional Development Planning Agency, still bears an annual lease obligation of approximately 6.7 billion Rupiah in 2025. In terms of revenue, the 2024 realization reached only 122,000,000 Rupiah, or 18.82 percent of the projected potential of 811,000,000 Rupiah. This imbalance is further illustrated by tourism data from the Bukittinggi Tourism Office and Telkomsel (January–September 2025), which recorded only 26,683 domestic visitors to *Lambuang* compared with 3,826,789 visitors to the nearby *Jam Gadang* area. These figures illustrate a paradox in which an ambitious physical development project generates new forms of dependency and weakens local economic agency due to strict enforcement by the Civil Service Police Unit responsible for public order regulations, combined with limited institutional coordination (Figure 1).

Previous studies consistently emphasize that the survival of small traders often depends on adaptive agency expressed through community solidarity (Bandauko, 2025; Malasan, 2019), as well as everyday resistance strategies (Bandauko, 2025; Recio, 2021), aimed at confronting spatial exclusion in urban environments (Onodugo et al., 2016). Interactions between traders and authorities tend to be negotiative and political in nature, often described as street entanglements shaped by micro-level political dynamics (Recio, 2022; te Linlo, 2017). Collectively, these studies focus on how agency navigates spatial conflict and legal ambiguity in urban contexts (Chan, 2018; Lin, 2018).

This study seeks to address a gap in the literature concerning how traders’ agency operates within formal markets that experience institutional stagnation after the completion of development projects. Through the concept of Forced Resilience, the research explores survival mechanisms in which traders actively reproduce the existence of a failing structure because they are legally and economically locked into it. Unlike the condition of exception typically associated with informal spaces, Roy (2005) forced resilience emerges when the state maintains legal authority while failing to provide adequate functional support for the infrastructure it has created. This study therefore provides a deeper analysis of how costly physical structures can shift from being development resources into sociological burdens that compel economic actors to subsidize the functional failures of the state in order to preserve the legality of their businesses.

## 2 Theoretical framework

### 2.1 Structuration theory and the duality of agency structure

Structuration Theory grounded in the core assumption of the duality of structure (Giddens, 1984). This principle rejects the traditional dichotomy between external forces and individual action. Within this framework, structure that includes institutions and rules and agency that refers to human action are not treated as separate. Instead, they are mutually constitutive and continuously reproduced through social practices. Structure functions as a dual modality that simultaneously constrains human action through normative rules while also enabling action through the allocation of resources. Human agency is assumed to be reflexive and capable of evaluating situations and acting autonomously. Through the mobilization of projective agency actors become essential elements in the reproduction of social systems and possess the capacity to create change or challenge existing institutional arrangements (Giddens, 1984).

Contemporary studies in economic geography consistently emphasize the importance of placing agency at the center of analyses of bottom up structural change (Grillitsch and Sotarauta, 2025). This body of literature highlights that agency must be understood within specific cultural and geographical contexts. Research demonstrates that public policy and entrepreneurial agency are essential in overcoming structural barriers (Holmen and Fosse, 2017). Three main types of transformative agency are widely regarded as key drivers of regional structural change. These include Schumpeterian agency institutional agency and leadership based agency (Benner, 2023; Grillitsch and Sotarauta, 2020).

Collectively these findings emphasize the complexity of agency which is shaped by both individual and collective assets that align with the concept of capital including economic cultural and social capital (Blažek et al., 2024). Agency operates within conditions of spatially bounded rationality (Huggins and Thompson, 2019). This condition is characterized by dynamic structural constraints in which agency strategies are limited by multiscalar power relations (Görmar et al., 2023). Consequently this situation calls for an integrated framework of agency that goes beyond the corporate level toward systemic agency including the conceptualization of governance entrepreneurship (Blažek and Květoň, 2023; Döringer, 2020). Actors may also assume roles that do not reflect formal hierarchies and instead deviate from narrowly defined economic interests (Grillitsch and Asheim, 2024).

### 2.2 Duality of structure in policy implementation and regional development

Studies on regional development typically focus on the prerequisites for success by emphasizing the need to balance agency and structure (Sotarauta and Beer, 2017). This perspective forms the basis for the role of place-based leadership, which Herzog and Hamm (2021) identify as a key driver of transformation. The literature also identifies several risks of failure. One important example is the phenomenon of inconspicuous maintenance, in this situation, the complexity of existing institutions can hinder innovation (Zietsma et al., 2018). Failure may also be intensified by the temporal nature of leadership intentions (Grillitsch et al., 2022) and by the fragmentation of institutional roles (Goodman, 2019). Provide a critical framework for understanding the potential failure of transformative sociotechnical transitions in large scale development projects (Schot and Steinmueller, 2018).

This study departs from the prevailing optimism within existing literature by positioning the *Lambuang* Culinary Area as an extreme case of transformative failure. The study argues that the failure of *Lambuang* represents evidence of institutional inertia that ultimately produces structural inertia. The primary contribution of this study lies in its sociological differentiation from other regional development studies. It reveals the mechanisms through which formal legality transforms from a facilitating instrument into a mechanism that reinforces structural inertia. Such analytical perspectives remain rare in development studies in Southeast Asia. These findings indicate that project failures in the region are not merely budgetary problems but reflect deeper sociological institutional traps.

### 2.3 Informal sector agency against structural stagnation

The literature on informal sector agency outlines various tactics of resistance and efforts to articulate marginalization through social networks (Adama, 2020; Alford et al., 2019). However these adaptive strategies remain vulnerable to structural pressures. Policies of spatial containment can undermine the organic characteristics of informal trade such as mobility and proximity to customers (Battisti et al., 2025; Kamalipour and Peimani, 2019). Within coercive environments traders are often forced into difficult compromises including strategic alliances with local coercive agents (Lata et al., 2019). Such conditions weaken collective agency and reduce the ability of actors to create new economic pathways (Grillitsch and Asheim, 2024; Huggins and Thompson, 2023).

This study differs from the prevailing narrative of heroic resilience. It introduces the concept of Forced Resilience which is defined as the sociological consequence of coercive structural failure. In the case of culinary traders in *Lambuang* agency is compelled to survive because actors are legally trapped without rational alternatives rather than being driven by proactive collective motivation. This condition states that the state often creates exceptional conditions through regulatory mechanisms (Roy, 2005). The study therefore provides a theoretical contribution that explains how legality and coercion can produce conditions of forced resilience which ultimately intensify individual atomization in cities of the Global South that struggle with failed infrastructure projects.

### 2.4 Conceptual framework: the structuration of forced resilience in failed development projects

This conceptual model illustrates a dialectical process in which structural dysfunction generates the conditions for the emergence of forced resilience. The process begins with structural deformation within a physical megaproject that experiences institutional paralysis in both management and land lease financing. This condition creates a state of exception, in which the government deploys its legal authority as an instrument of dominance to suppress organic street economies (Roy, 2005). As a result, spatial containment occurs, leading traders to experience legal and economic lock-in. This represents a defining operational boundary that distinguishes this case from ordinary market failure, as coercive threats prevent actors from returning to informal spaces through rigid spatial regulations.

At the subsequent stage, the framework demonstrates the operation of the duality of structure as conceptualized by Giddens (1984). Forced resilience emerges not as a voluntary adaptation but as a defensive response by traders seeking to maintain legal legitimacy within a dysfunctional structure. In this process, traders mobilize their social capital to subsidize the failure of state service provision so that the market continues to appear formally operational. From a theoretical perspective, these survival strategies constitute recursive practices that ironically reproduce and sustain the existence of the failing project structure in order to preserve legitimacy and ensure the continued survival of the actors involved.

## 3 Method

This study employs a qualitative approach with a case study design to examine the dialectical relationship between policy structures and the agency of traders in the *Lambuang* Culinary Area of Bukittinggi, West Sumatra, Indonesia (Yin, 2018). This design was selected to uncover the social processes underlying the failure of a megaproject by applying Structuration Theory (Giddens, 1984) as the primary analytical framework for identifying the emergence of Forced Resilience. The research site was chosen because it represents an extreme megaproject paradox, where financial failure and managerial paralysis have created tangible structural burdens for micro-scale economic actors.

Research participants were selected using purposive sampling to ensure a balanced representation of both structural and agency dimensions (Creswell and Poth, 2016). The composition of informants was strengthened by involving three local government authorities representing the regional planning unit as well as management and technical supervisory units, allowing the study to capture the operational logic behind formal policy decisions. Simultaneously, nine informants consisting of traders and visitors were included in order to document survival strategies and the sociological consequences of market failure. The representation of the 12 key informants is presented in Table 1 as the basis for source triangulation, which was used to validate the phenomenon of legal entrapment experienced by the research subjects.

**Table 1:** Information about participants.

Code	Category	Gender	Age (years)
Informant 1	Vendor	Male	40
Informant 2	Vendor	Female	45
Informant 3	Vendor	Female	46
Informant 4	Local government	Male	57
Informant 5	Vendor	Male	59
Informant 6	Vendor	Female	52
Informant 7	Vendor	Male	32
Informant 8	Visitor	Female	20
Informant 9	Vendor	Male	55
Informant 10	Local government	Male	59
Informant 11	Vendor	Female	52
Informant 12	Local government	Male	44

Data were collected through in-depth interviews using a semi-structured protocol. In response to methodological transparency requirements, the interview instrument was designed based on the three core domains of structuration. The first domain, signification, explored traders’ understanding of formal market regulations. The second domain, domination, examined the allocation of resources and the exercise of authority, particularly through the enforcement role of the Civil Service Police Unit. The third domain, legitimation, analyzed legal norms and rental obligations within the market system. Key interview questions addressed the reasons why traders continue operating within a dysfunctional market environment, the challenges they face in complying with regulatory requirements, and the role of informal capital in coping with operational cost pressures.

Data integrity was maintained through first author reflexivity, positioning the researcher as a key yet objective instrument capable of minimizing subjective bias while still capturing local terminologies such as *hidup*, which refers to defensive survival within constrained economic conditions. Primary interview data were further verified through methodological triangulation with secondary sources, including official documents related to the investment budget of approximately 24.5 billion Indonesian Rupiah and the fiscal burden of land lease payments totaling 9.7 billion Rupiah. This triangulation ensured that theoretical interpretations remained grounded in empirical material evidence.

Data analysis followed the interactive model proposed by Miles et al. (2014), which consists of data reduction, matrix-based data display, and conclusion drawing. The analytical procedure was conducted in a layered manner by clearly distinguishing between first-order descriptions, representing the authentic narratives of informants, and second-order interpretations, which reflect the researcher’s theoretical construction. Through pattern coding, the concept of Forced Resilience was operationalized by mapping how structural constraints recursively encourage agency to reproduce dysfunctional structures. The validity of the final findings was further ensured through a member checking procedure, confirming that the conceptual formulation presented in this study accurately reflects the sociological dynamics observed in the field.

## 4 Results

### 4.1 Survival strategies within a legal entrapment

**Figure 2:**
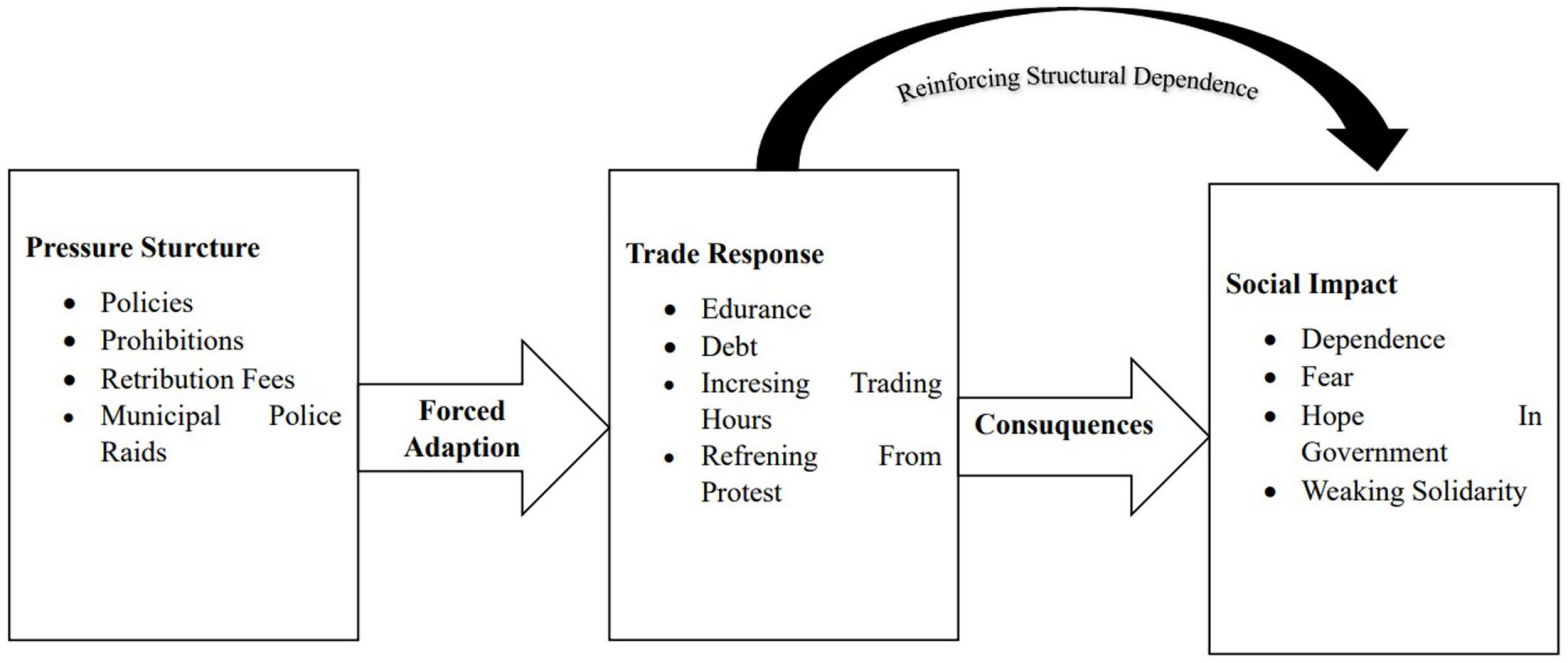
Forced resilience cycle of vendors in the *Lambuang* street food area under structural pressure. Source: Researcher’s findings, 2025.

Figure 2 illustrates the collective cycle of forced resilience experienced by traders in the *Lambuang* Culinary Area. The core of this cycle demonstrates that structural pressures originating from restrictive policies, rising operational costs, and strict enforcement by the Civil Service Police Unit generate survival responses that do not arise from voluntary agency but rather from coercion. Ironically, each survival strategy adopted by traders reinforces their dependence on the failing system while simultaneously weakening their economic autonomy. In this context, resilience does not represent strength or successful adaptation but instead reflects a painful form of adjustment within a structure that refuses to provide sustainable alternatives for livelihood. Informant 1 highlighted the stark contrast between past and present conditions, emphasizing the dramatic decline in daily income:

“…When I first started selling here, the place was crowded and I could earn up to IDR 2,000,000 a day. Now there are very few buyers, and sometimes I only make IDR 250,000 in a full day. Even though it is difficult, we are forced to stay because there are no other options. We cannot sell outside, so we remain here even when the market is empty. If we leave, we would have no income at all…” (Informant 1, interview, 31 October 2025).

The repeated use of the phrase “forced to stay” becomes central to the analysis, indicating that traders have effectively lost viable structural alternatives under rigid regulatory conditions. Field findings regarding the perception of having no other choice reflect both psychological and economic entrapment. A market that would normally be abandoned due to low demand cannot be left because legal restrictions prohibit trading elsewhere. As a result, the *Lambuang* area has gradually transformed from a site of profit generation into a space primarily dedicated to risk mitigation and daily survival.

This sense of entrapment is further intensified by unavoidable operational costs. Informant 2 described the dilemma between structural obligations and declining revenue:

“…I open my stall from the evening until dawn and even add new menu items as a survival strategy. If the losses continue, I may have to move. I do not want to keep losing money because rental costs and product prices keep rising while income continues to decline…” (Informant 2, interview, 31 October 2025).

Economically, this statement highlights an impossible trade-off. Traders actively attempt to adapt by extending operating hours and modifying their menus. However, these individual efforts collide with escalating structural pressures such as rent obligations and rising product costs. The pursuit of economic independence becomes illusory when structural interventions that should support the market, such as promotional programs or rent adjustments, remain absent. Culinary traders who once symbolized the cultural identity of the city now operate in an environment where the market has lost its social vitality.

The struggle for survival ultimately leads to more severe financial consequences. Informant 3 revealed one of the most vulnerable dimensions of the forced resilience cycle: the emergence of new economic traps.

“…I stay here because there is nowhere else to go, and I must follow the rules enforced by the Civil Service Police Unit that crack down on street vendors. I used to earn about IDR 2,000,000 in gross income, but now it is difficult to make even IDR 500,000. I was forced to borrow money from informal lenders just to have capital for my children’s daily needs…” (Informant 3, interview, 30 October 2025).

These findings demonstrate how structural regulatory pressure has evolved into existential financial pressure. In order to meet basic needs amid declining revenue, traders are compelled to rely on destructive informal financing sources such as loan sharks. This coping strategy further deepens their vulnerability and dependency. Consequently, their resilience is no longer measured by the ability to generate profit but rather by their capacity to absorb and endure accumulating debt burdens.

### 4.2 From policy design to the production of public issues

**Figure 3:**
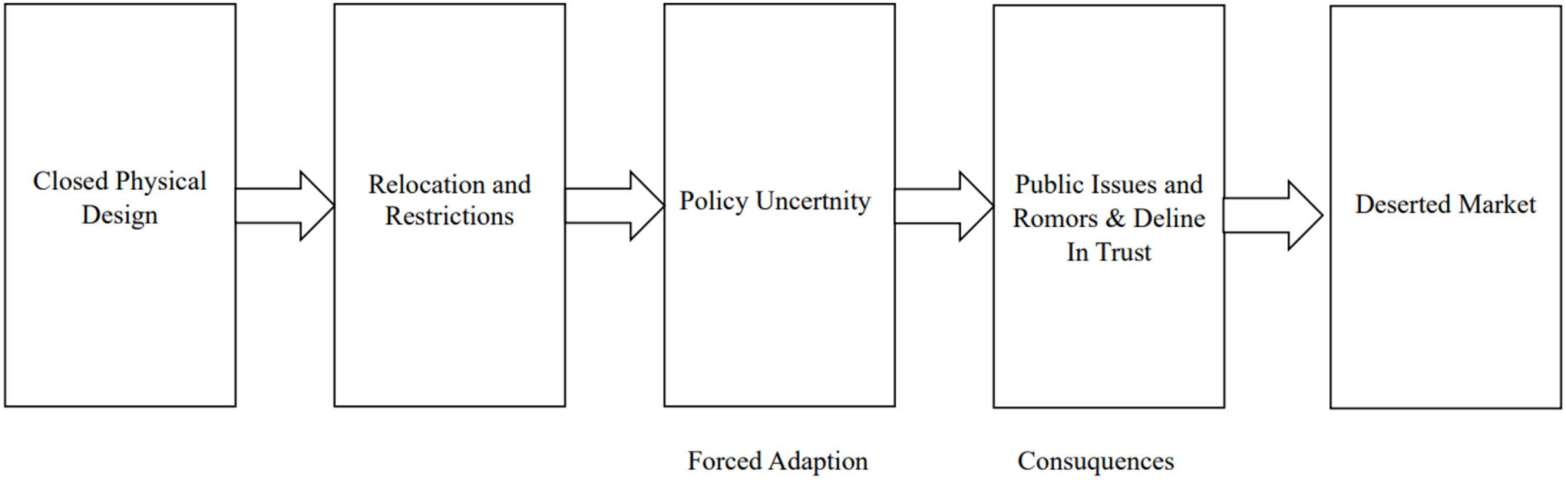
The chain of decreasing crowds: From policy design to public issue production (Source: Researcher’s findings, 2025).

The sharp decline in the number of visitors in the culinary area is not an isolated event but the result of a locked structural chain as illustrated in Figure 3. This failure demonstrates a fundamental dissonance between the macro development vision focused on urban aesthetics and the micro social reality of market habitus.

Figure 3 illustrates the chain of visitor decline. The failure to transform physical capital into social capital became the initial trigger of the trust crisis. This structural mismatch is confirmed by planning authorities who acknowledge that the project was driven more by the logic of urban cleansing than by functional market studies. Through the lens of Structuration Theory, this statement reveals a gap in structural signification. The government framed the project as a moral and aesthetic necessity but failed to provide allocative resources such as operational details and sustainable budget planning. Informant 12 clarified the macro aesthetic vision behind the initiation of the project.

“…The local government saw the old station area as very concerning. We even found used condoms there. We were worried the place could be misused and damage the image of the city. The Regional Planning Agency works at the macro level. We requested priority programs from the regional leader to be included in the Regional Development Plan. However the detailed planning and budget research were the responsibility of the technical departments…” (Informant 12, interview, 30 October 2025).

Based on the condensation of research data, the forced resilience experienced by vendors is a direct consequence of policies imposed from above that prioritize urban image over the sociology of markets. The planning process, although formally documented, failed to consider the open space trading habitus in Bukittinggi. As a result the physical structure functions more as a cage than as an economic resource.

**Figure 4:**
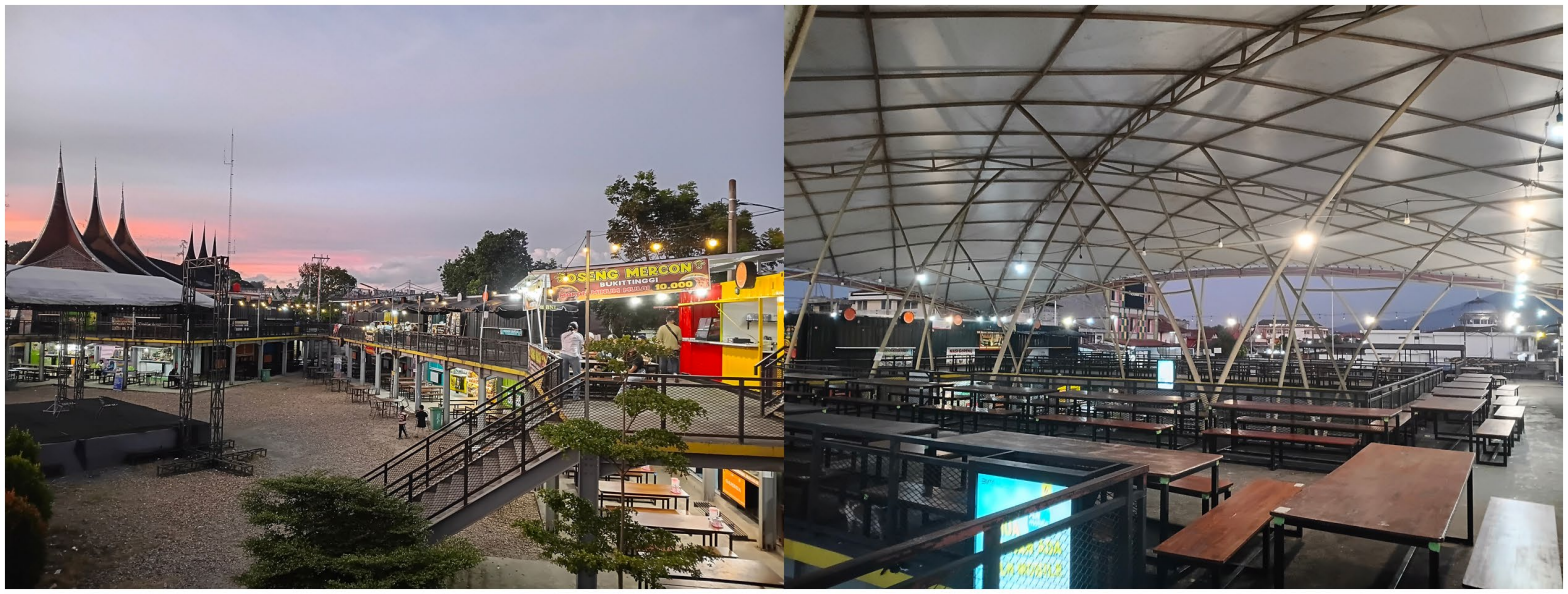
Condition of the street food area *Lambuang* in the inner part. Source: Researcher’s findings, 2025.

Figure 4 shows the internal condition of the *Lambuang* Culinary Area. This visual evidence demonstrates that the absence of visitors cannot be explained simply by fluctuations in public purchasing power. Instead the condition reflects the accumulation of a chain of structural failures. The most fundamental structural problem lies in the contradiction between the physical design of the project and the social reality of the local community. Informant 4 explained that the functional failure of the market is closely related to architectural design problems.

“…The occupancy rate is low at around twenty five percent of the one hundred and sixteen kiosks that were built because the physical design does not match social reality. The building is surrounded by fences and the access routes are complicated and hidden. These conditions do not encourage people to enter. People in Bukittinggi prefer open spaces such as sidewalks rather than closed concepts like this…” (Informant 4, interview, 28 October 2025).

These findings reinforce that the project represents an extreme case of design adaptation failure. Instead of responding to the consumption habits and patterns of social interaction within the local community, the design created an exclusive and socially detached structure. The fenced and hidden buildings effectively disconnected the market from the social rhythm of the city and produced structural alienation that eventually resulted in financial failure.

**Figure 5:**
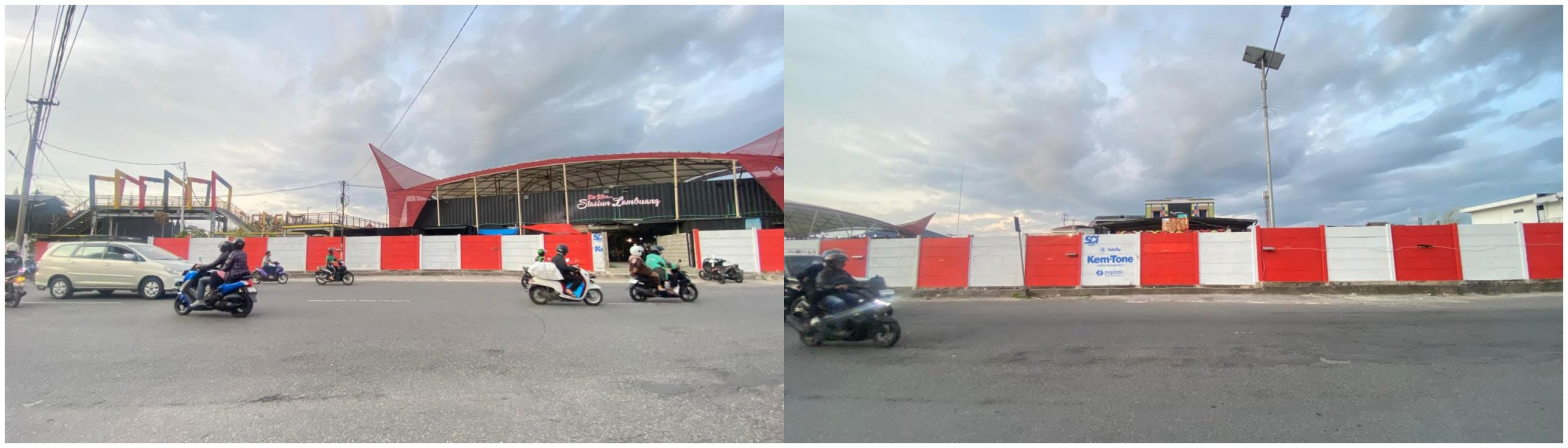
Condition of the street food area *Lambuang* in the outer part (Source: Researcher’s Findings, 2025).

Figure 5 presents the boundary fence and the external access area. The quiet condition outside the complex demonstrates that when public space is fenced and separated from the social circulation of the city, economic activity also declines. This situation reflects what Ananya Roy describes as urban informality where the state formalizes space in ways that suppress the economic logic of grassroots traders. This finding is reinforced by the experience of Informant 5 who described the paradox of legality.

“…We were long time vendors who were directed to move from the roadside to this location. The kiosks were allocated through a lottery and facilities were provided. We are officially registered so we must follow the rules. We cannot return to the street because we are afraid of enforcement actions by the Civil Service Police Unit…” (Informant 5, interview, 15 October 2025).

This finding adds an important structural dimension. Forced relocation disrupted the previous social and economic networks of vendors. Formal legality which should function as protection has instead transformed into a structural trap. Compliance is enforced in a context of uncertainty which locks vendors into a system that demands obedience without guaranteeing economic welfare. This trapped condition becomes more severe due to policy dynamics and changes in local political leadership that introduce new forms of uncertainty.

**Figure 6:**
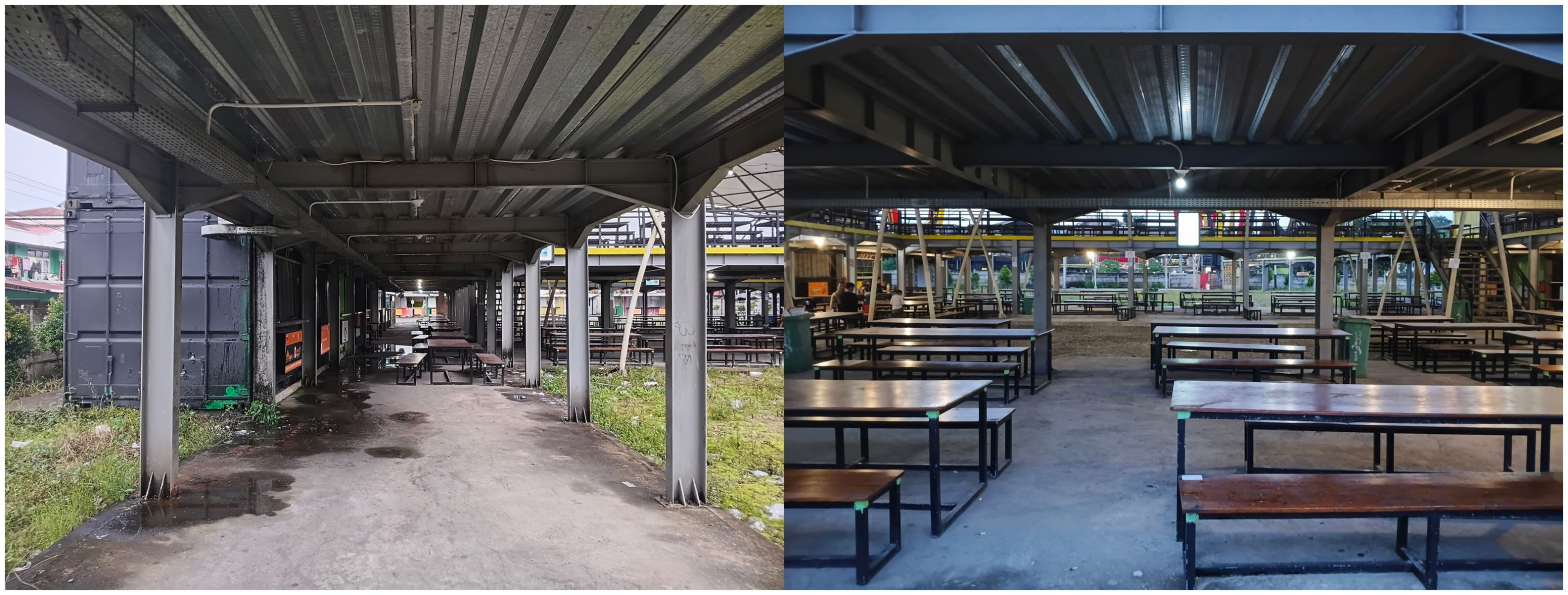
Neglected kiosk condition and empty dining table area (Source: Researcher’s findings, 2025).

Figure 6 illustrate abandoned kiosks and empty dining areas. These visuals show how uncertainty spreads into the policy environment and public perception and makes the market condition increasingly fragile. Informant 6 explained that the problem involves not only an unfriendly social space but also the circulation of public rumors and changes in government leadership.

“…This place has been quiet since rumors spread that the market will close. Those rumors made buyers afraid to come. There was also a change of mayor who does not want to extend the contract so we feel abandoned even though the facilities still exist and we continue paying rent every month…” (Informant 6, interview, 3 November 2025).

This statement shows that problematic design intersects with political policy uncertainty and intensifies the negative impact of relocation. Changes in power and a hands off attitude from the local government create a situation in which vendors remain bound within a legal system that demands compliance through rent payments but does not guarantee economic sustainability. Public statements without concrete follow up actions can undermine market confidence. The domino effect of inconsistent policies places vendors under dual pressure from internal design problems and from the unclear direction of authorities.

**Figure 7:**
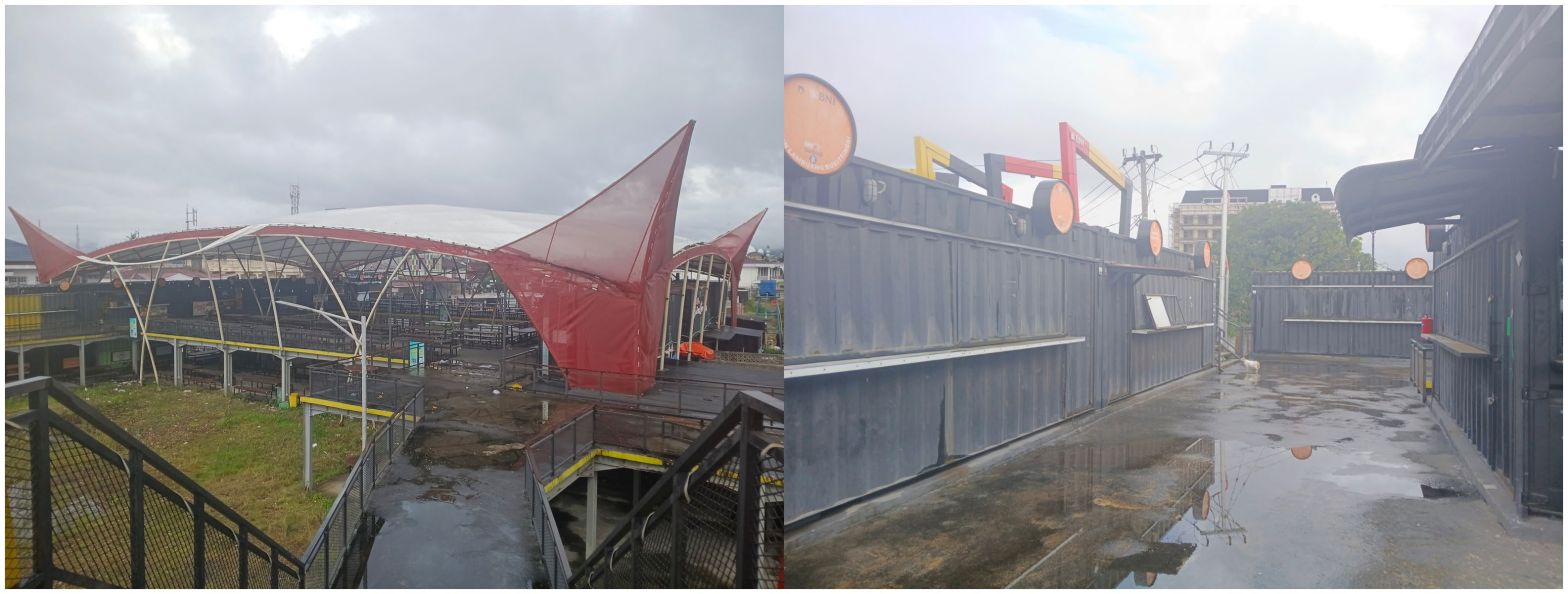
The iconic rooftop is deserted and the container hallway is empty (Source: Researcher’s Findings, 2025).

Figure 7 show the quiet iconic roof area and the empty container corridors. Visitor perspectives further strengthen the finding that rumors may have influence equal to or even stronger than official policies. Projects that originate from the vision of a particular political leader become vulnerable when leadership changes. Informant 7 emphasized that negative perceptions spread rapidly and produce a new social reality based on distrust.

“…The market is not empty because people are not interested. The problem is the lack of clarity from the government and the absence of promotion. There are many confusing rumors about management which make visitors hesitant to come…” (Informant 7, interview, 3 October 2025).

These findings highlight that the emptiness of the culinary area is largely shaped by failures in government communication. While the public waits for certainty the information circulating in society consists mainly of contradictory rumors. Overall the findings lead to a clear conclusion. When structural policies become inconsistent and public narratives are not managed effectively the market loses its social legitimacy. The decline in visitors is the result of a locked structural chain consisting of misguided spatial design forced relocation that pressures vendors inconsistent policy directions and rumors that generate social fear. The culinary market is not simply quiet. It has become a reflection of the erosion of trust between citizens vendors and government.

### 4.3 Agency longing for structural commitment

**Figure 8:**
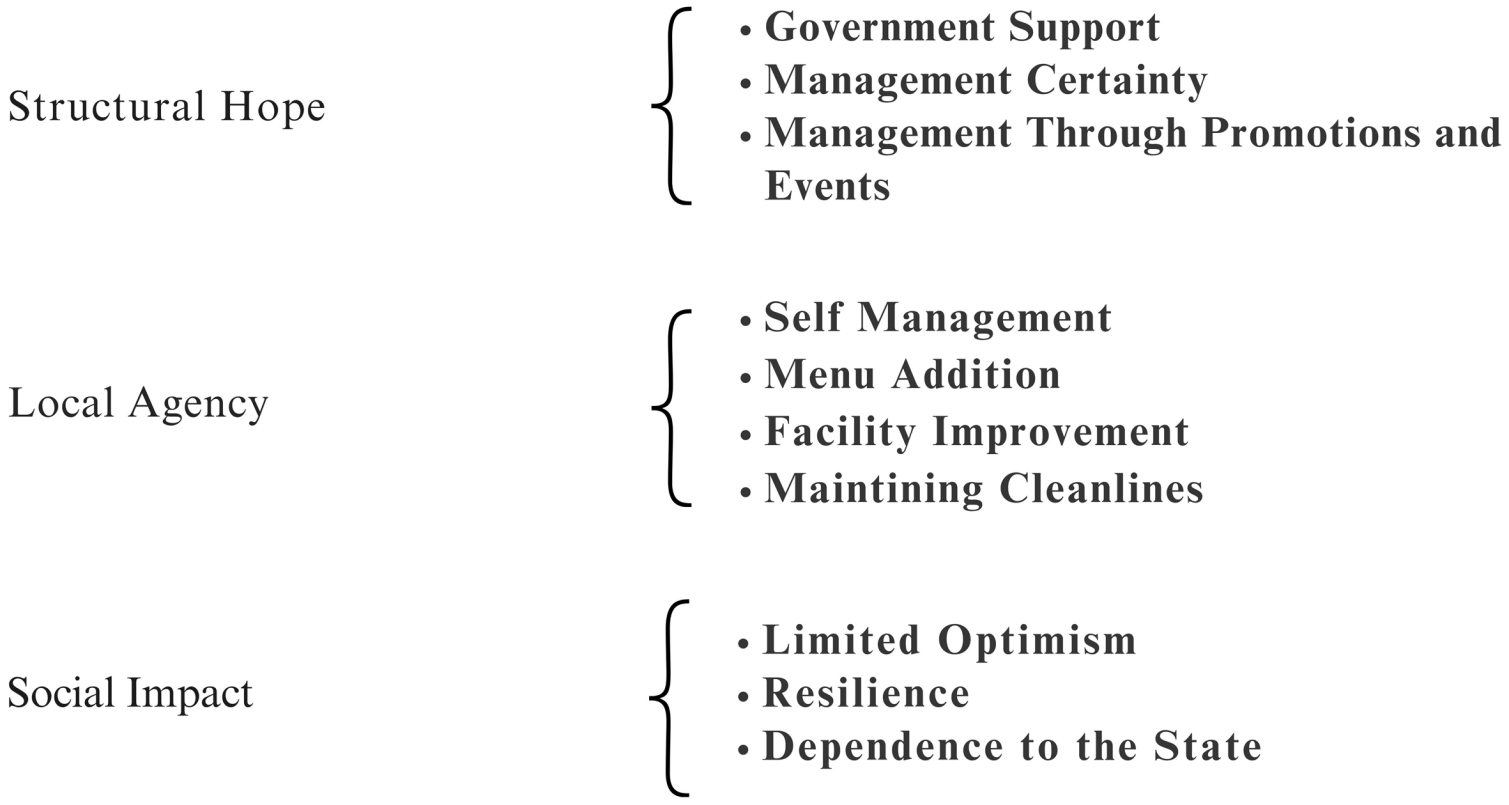
Patterns of hope and agency in trader recovery at *Lambuang* street food area (Source: Researcher’s findings, 2025).

The analysis in this section presents an in depth interpretation of the dynamics of the culinary market through the framework of empirical findings illustrated in Figure 8. This model examines how institutional weakness triggers conditions of forced resilience through three interconnected layers. These layers consist of structural expectations, local agency, and social impacts.

Figure 8 illustrates the pattern of expectations and agency in the recovery of vendors. Structural inertia driven by fragmentation and the discontinuity of political commitment explains the contradiction between the expectations of agency and the empirical reality in the field. Institutional evidence shows a neglect of vendors’ expectations regarding the security of market management. Informant 10 stated that the failure of this project was caused by macro level policy rather than stagnation at the micro level.

“…This land belongs to the State Railway Company so the local government only rents it. Because the company operates with a business orientation the land lease for five years reached around Rp9.7 billion. The project initiated by the mayor for the period 2021 until 2025 involved the Planning Agency and several local departments. However the local government cannot continue the lease until 2027 because of the weak regional fiscal condition and the decision of the newly elected mayor for the period 2025 until 2030…” (Informant 10, interview, 27 October 2025).

This structural inertia operates across multiple scales. The empirical failure of the project can be explained by three main factors which include conflicts of interest between the local government and a state owned enterprise, fiscal limitations, and the disruption of project continuity due to changes in political leadership. From the perspective of Giddens’ Structuration Theory this political discontinuity causes the structure to lose its role as a provider of enabling resources and instead transforms it into a coercive burden that obstructs revitalization. The response of local agency to this inertia takes the form of survival strategies and self-management initiatives. Structural pressure initially triggers mechanisms of social exclusion that are experienced by consumers. Informant 8 stated the following.

“…High prices which result from expensive rental costs make students reluctant to come. We hope the government can improve lighting and cleanliness, introduce local menus, and organize regular events. If the prices are friendly for students this place could become lively again…” (Informant 8, interview, 6 November 2025).

This statement demonstrates that structural pressure caused by rental costs directly damages the function of the market as an inclusive social arena. The exclusion of students as a key consumer group represents clear evidence of structural failure in creating a sustainable ecosystem. From the perspective of vendors the response to political pressure takes the form of strategic silence as explained by Informant 9.

“…We choose to remain silent and passive while waiting for instructions so that we are not seen as resisting. The most important thing is to stay safe and avoid eviction. We only hope that this place will not be abandoned and that it will be promoted so people know it is still open…” (Informant 9, interview, 31 October 2025).

This statement shows that the agency of vendors operates in a rational mode that aims to minimize the risk of eviction. Limited resources encourage the emergence of self management as a form of collective micro level action that attempts to fill the gap in basic services. Informant 11 stated the following.

“…In the past we received assistance for clean water but now the machine is broken and we must bring water ourselves. Electricity and cleanliness are now managed by the vendors themselves. We only hope the government will promote this place again…” (Informant 11, interview, 26 October 2025).

From a sociological perspective this form of local agency functions only as a temporary response to the deficits left by an absent structure. Although these actions create resilience that allows the market to continue operating this form of agency is essentially coercive. Vendors are forced to redirect their own resources to cover the costs of basic management that should be the responsibility of public authorities. This phenomenon reflects what Ananya Roy describes as planned neglect where institutional structures continue to demand compliance through rent obligations while failing to provide reciprocal services. The entire cycle culminates in a condition of forced resilience. Through this mechanism the self-management efforts of vendors paradoxically reproduce the very structure that oppresses them. Vendors continue to repair the shortcomings of the system so that the failing system appears to function. The culinary area therefore stands as evidence of structural failure that forces agency to survive under conditions of dependency and atomization which ultimately obstruct the possibility of sustainable territorial transformation.

## 5 Discussion

The case study conducted in the *Lambuang* Culinary Area of Bukittinggi indicates the presence of obstacles in the development process that can be identified as structural inertia. This condition emerges when the institutional capacity of the local government stagnates in managerial and financial aspects, thereby transforming the function of the project from a facilitating instrument into a structure that tends to impose constraints. Empirical data show that traders occupy a legally difficult position because they are effectively locked into a physical complex that is economically unproductive while lacking access to alternative formal spaces. This situation has encouraged the emergence of forced resilience, in which traders’ survival strategies represent responses to legal and economic risks rather than proactive entrepreneurial choices.

The entrapment of agency within a poorly functioning structure provides an additional perspective on Structuration Theory (Giddens, 1984). In this context, the structure of legality appears to function more as a constraint than as a provider of economic resources for agency. The researchers observed that although the physical facilities failed to attract visitors, traders continued to perform recurring social practices such as fulfilling administrative obligations in order to maintain business legality. These recursive practices indirectly contribute to sustaining the existence of a system that is experiencing functional difficulties. This observation aligns with the view that agency plays a crucial role in structuration dynamics at the regional level (Grillitsch and Sotarauta, 2025; Gruchmann et al., 2019; Recio et al., 2017).

When compared with studies of informality in other contexts, such as Harare (Bhila and Chiwenga, 2023) and Hong Kong Kong (Smart and Smart, 2017), which emphasize physical repression, the case of Bukittinggi demonstrates a different dynamic. The primary challenge faced by traders does not stem from aggressive regulatory enforcement but rather from functional neglect within an already formalized space. The study argues that the process of formalization implemented by the local government tends to create conditions that restrict the organic economic flexibility of traders. This finding resonates with discussions on urban informality, which highlight how planning processes may produce spaces that are misaligned with the operational logic of grassroots markets (Roy, 2005; Song, 2016; Zietsma et al., 2018).

Research on regulatory negotiation in Bangkok by Batréau and Bonnet (2016) suggests a more optimistic outlook regarding the potential for policy dialogue. However, such expectations appear difficult to apply in the *Lambuang* context. Field observations indicate that compliance with legal requirements in formal spaces often involves costs that are disproportionate to the income generated. The researchers found that this imbalance encourages some traders to rely on informal financial support to cover daily operational expenses. These findings support previous discussions on the risks associated with formalization processes that are not accompanied by adequate functional support for small-scale economic actors (Huggins and Thompson, 2023; Koch, 2015; Linares, 2018; Liu et al., 2025; Mramba, 2025).

The study also observes that the lack of inclusive dialogue following the crisis in Bukittinggi has slowed the process of market recovery. The absence of two-way communication between authorities and economic actors reinforces institutional barriers that are commonly found in large-scale development projects. The researchers therefore recommend the implementation of more adaptive policy interventions to repair the development trajectory of the project so that economic actors are not continually trapped within structural limitations to economic access (Huggins and Thompson, 2019; Ibrahim et al., 2024; Nilsen et al., 2023).

As a theoretical contribution, this study proposes the concept of Informal Governance Entrepreneurship. The term refers to the collective efforts of traders to manage facilities independently when public services fail to function effectively. These actions can be interpreted as a form of agency that seeks to sustain the basic functions of the market despite limited structural support. This perspective helps explain how small-scale actors may temporarily act as institutional substitutes when formal systems experience governance stagnation (Adeosun et al., 2023; Blažek and Květoň, 2023).

Overall, this research provides an overview of how structural inertia affects the microeconomic ecosystem in Southeast Asia. Through the conceptualization of forced resilience, the study highlights the complex adaptive strategies adopted by traders in policy environments characterized by limited flexibility. These findings are expected to contribute to the broader discussion on more inclusive approaches to urban development in the future.

## 6 Conclusion

This study concludes that the failure of the public space megaproject in Bukittinggi represents a manifestation of structural inertia that transforms the function of policy from an instrument of empowerment into a sociological burden for small economic actors. The main findings indicate that the phenomenon of forced resilience compels agency to reproduce failing structures through recursive practices in order to maintain legal legitimacy. This demonstrates that agency is not passive; rather, actors engage in informal governance entrepreneurship to fill the gap left by the state’s limited role. The limitations of this study lie in its case study focus, which is closely tied to local political dynamics. As a result, the generalization of the findings should be approached with caution. In addition, limited access to internal financial data from the local government restricts the depth of the analysis regarding the technical dimensions of budget inefficiency. For future policy, a paradigm shift is needed from aesthetically oriented physical development toward planning grounded in social functionality and multi-stakeholder dialogue. As a next step, comparative studies across different regions of the Global South are necessary to examine patterns of structural inertia in diverse administrative contexts. Future research should also explore how digital technologies can be utilized by local agency as a new form of social capital in addressing governance deadlocks within formal public spaces.

## Data Availability

The raw data supporting the conclusions of this article will be made available by the authors, without undue reservation.
